# Tooth-Picks

**Published:** 1871-02

**Authors:** 


### TOOTH-PICKS.

A SENTENCE is going the rounds of the papers, which is most pernicious, that one tooth-pick is worth a bushel of tooth-brushes. The people must get into the habit of weighing every practical statement in reference to the health and well-being of our bodies, or they will be led by specious statements, or striking comparisons, or popular names, to many hurtful habits, practices, and observances. Every dentist knows that the more a tooth-pick is used, the more the yielding gum is pressed upward; and the larger the space becomes between the teeth next the gum, the larger will be the pieces of food that will lodge there, and the greater the necessity of the use of the tooth-pick for the remainder of life, and to some extent the solid tooth itself may wear away.

If the tooth is hollow, it ought to be extracted or filled with gold at the very earliest moment possible, so as to prevent decay and a noisome breath.

To be under the necessity of spending five or ten or more minutes after each meal in picking the teeth is a great and useless waste of time, and is essentially a loafing and indecent practice, unless done in one's own private room. On the other hand, the intelligent dentist knows that the judicious use of a tooth-brush immediately after each meal, twisting it up and down sideways, so that each bristle becomes a soft tooth-pick, and gently dislodges particles of food, while at the same time it hardens the gum, makes it more firm, causes it to grow more fully into any crevices, and fills them up more perfectly. Whether by pick or brush the same important end is sought, to keep the teeth clean, because it is the only means of preserving them, and the want of it the certain means of their speedy destruction in almost every case. The true statement of the proposition is to reverse it, and say that one tooth-brush is worth a cart-load of the indecent and always hurtful tooth-pick.

